# Supplementary material for: Fear, Efficacy, and Environmental Health Risk Reporting: Complex Responses to Water Quality Test Results in Low-Income Communities
Source: Int J Environ Res Public Health. 2022 Jan 5;19(1):597. doi: 10.3390/ijerph19010597 (PMC8744942; doi:10.3390/ijerph19010597)

Supplementary Figure S1: Association plot for perceived water safety by main drinking-water supply type. *Blue shading indicates that the observed value is higher than expected if the data were random, and red shading indicates that the observed value is lower than expected. The 'Other' category includes rainwater, bottled water, vended jerrican water, and water that is piped into a compound or household, delivered by a tanker truck, or borrowed from a neighbour.*

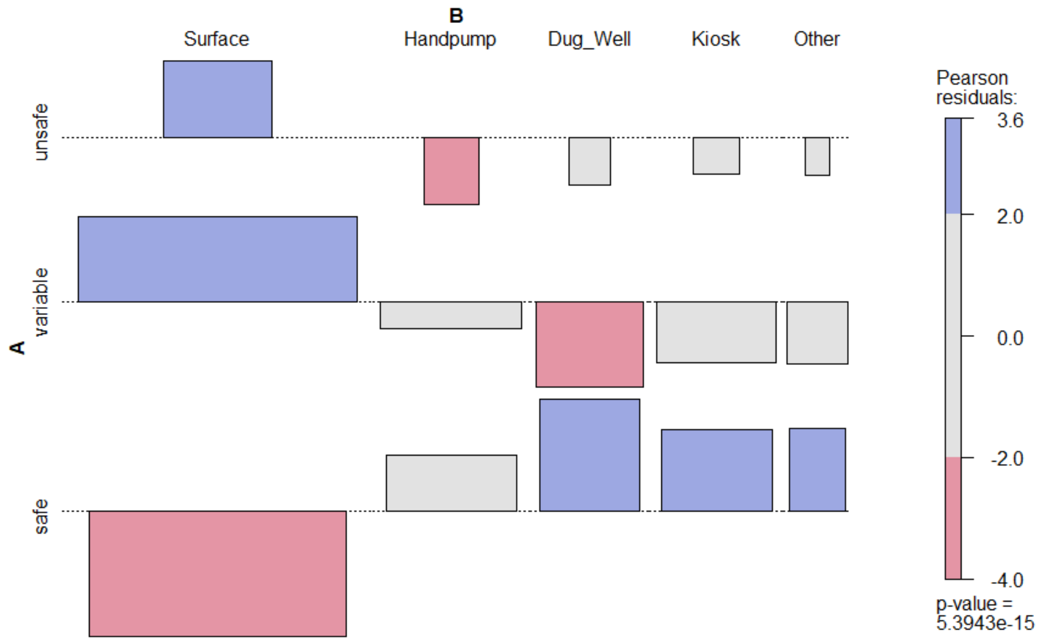

Supplementary Figure S2: Counts of reported stomach pain and diarrhoea by suspected cause from the longitudinal household survey. *Stomach pain was reported from 59 households with each household reporting between 1 and 6 times (mean = 2.3, median = 2). Diarrhoea was reported from 31 households with each household reporting between 1 and 6 times (mean = 1.7, median = 1).*

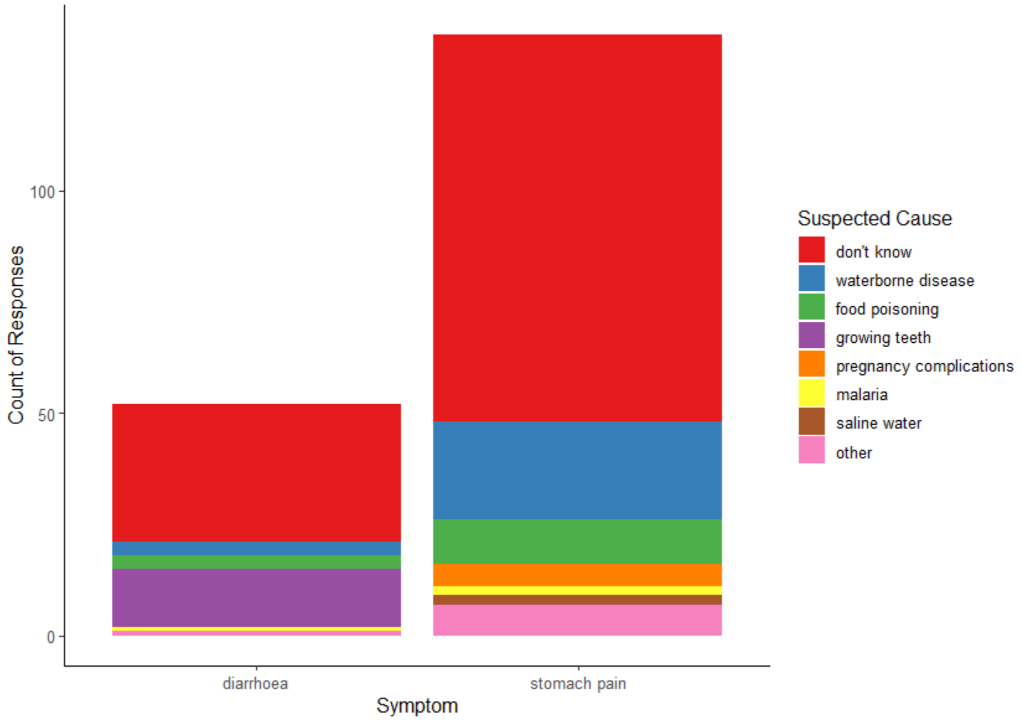

Supplementary Figure S3: Contribution biplot of affect display by change in perceived susceptibility.

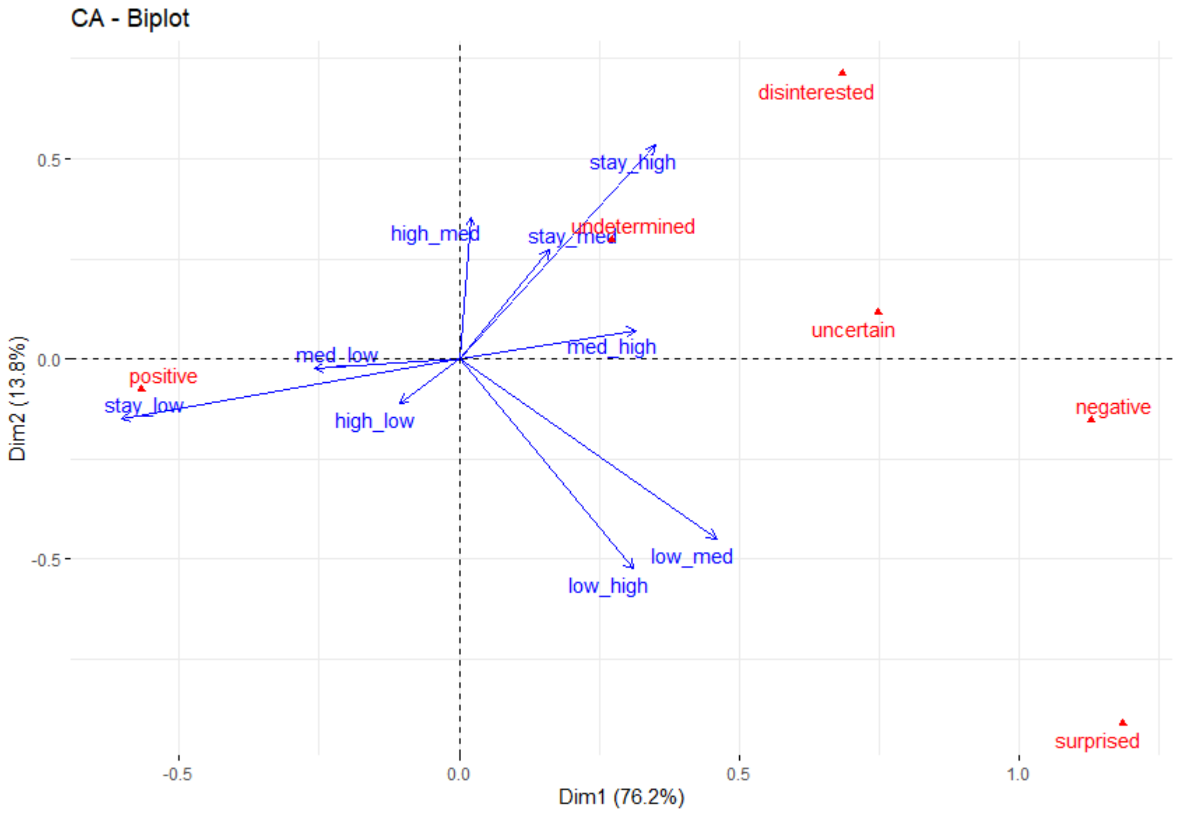

Supplementary Figure S4: Contribution biplot of stage of change by affect display.

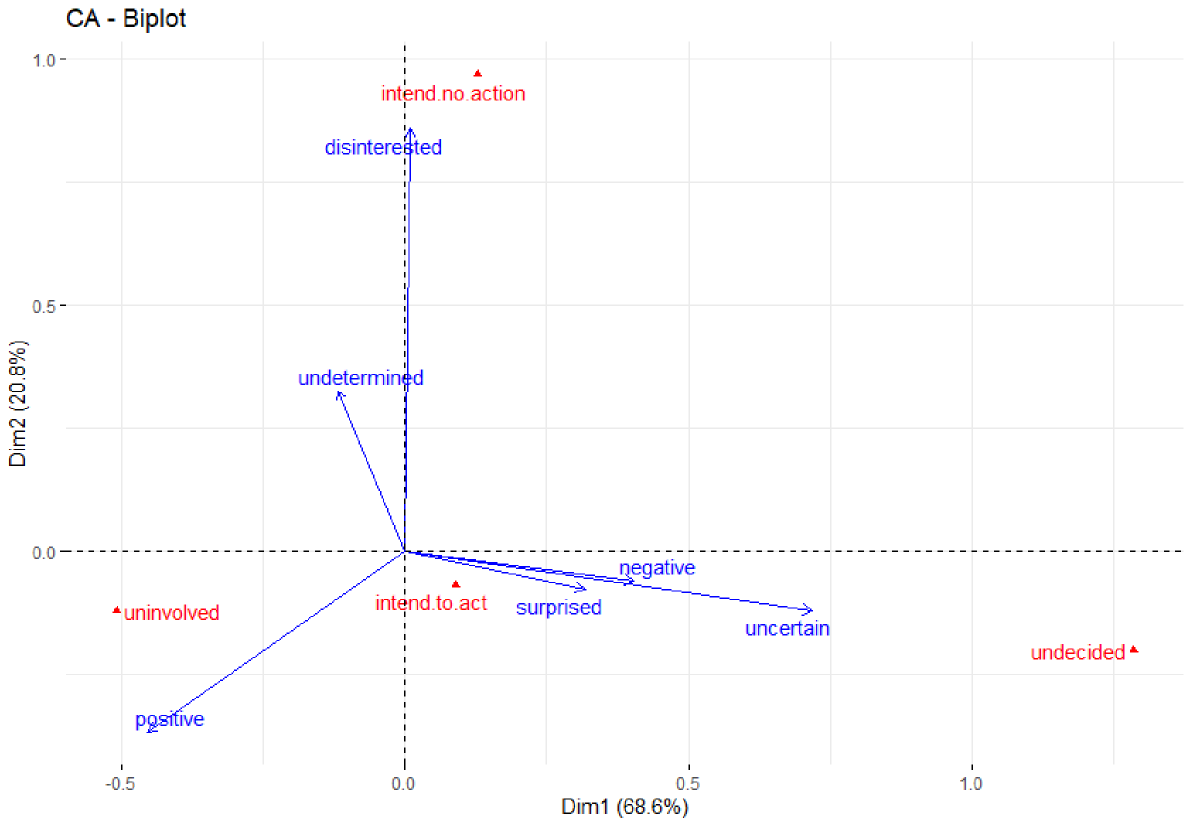

Supplementary Figure S5: LWM stages of change at each time step.

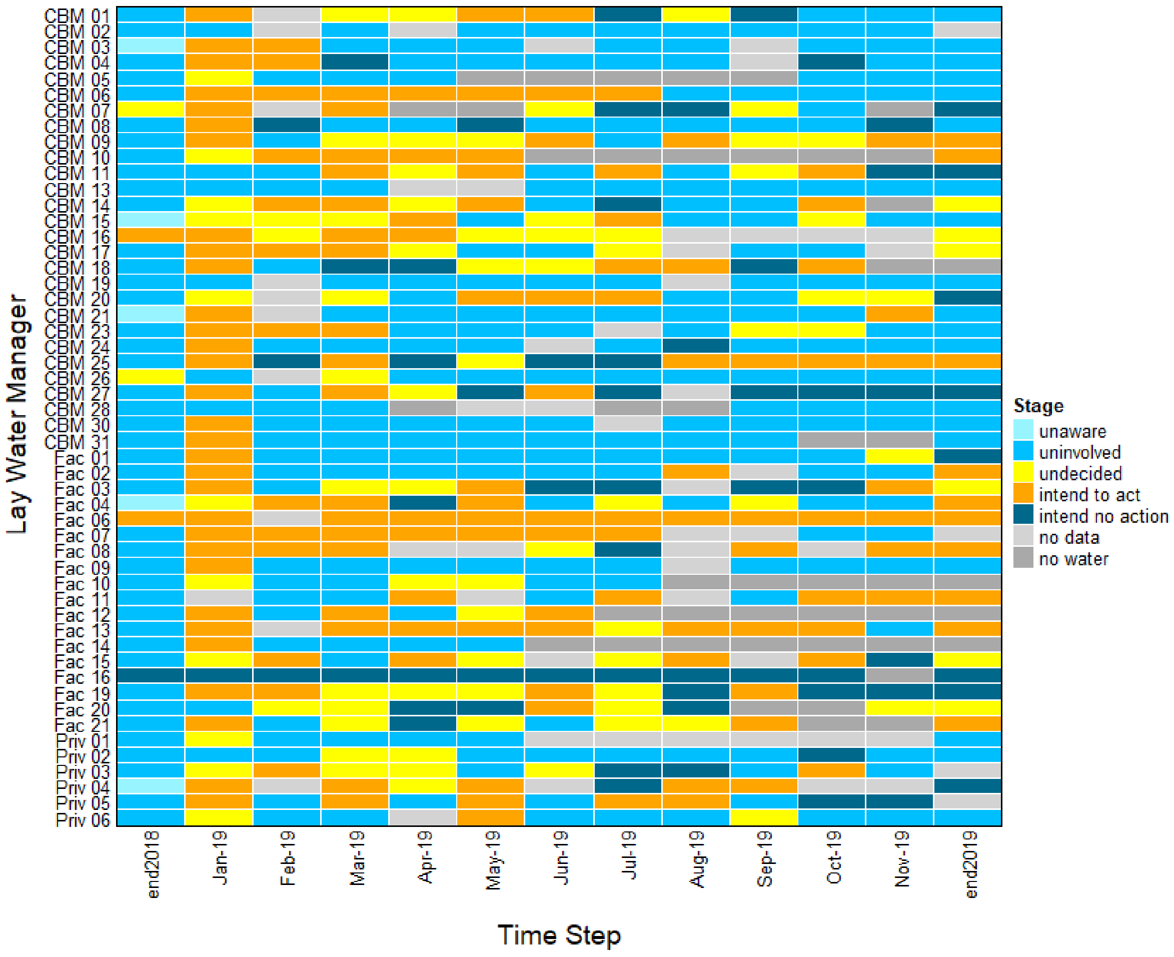

Supplement: Supplementary file 1 [file ijerph-19-00597-s001.zip › Supplementary_Figures.pdf]
