# Supplementary material for: Fear, Efficacy, and Environmental Health Risk Reporting: Complex Responses to Water Quality Test Results in Low-Income Communities
Source: Int J Environ Res Public Health. 2022 Jan 5;19(1):597. doi: 10.3390/ijerph19010597 (PMC8744942; doi:10.3390/ijerph19010597)
Supplement: Supplementary file 1 [file ijerph-19-00597-s001.zip › Supplementary_Files.pdf]

Supplementary File S1: Longitudinal Household Survey Check-in Questionnaire

The water and health check-in questionnaire was administered biweekly to complement the water diaries study. The questionnaire has two sections focussing on water source perceptions and health outcomes.

Section 1: Water Source Questions

	Question	Response	Notes
1	Did you purchase bottled or sachet water in the last two weeks?	Yes/No	Asking these upfront so that they are less associated with illness questions.
1a	**IF** yes, why did you buy it?	[text field]	
2	Have you done any water treatment in the last two weeks?	Yes/No	
2a	**IF** yes, what did you do?	[text field]	
2b	**IF** yes, why did you do it?	[text field]	
3	Have there been any problems with your water source(s) in the last two weeks?<br>[*select all that apply*] add no prompt caveat	a) No problem<br>b) Breakdown<br>c) Supply cut-off<br>d) Source is dry<br>e) Water quality issues<br>f) Other [text field]	This should be asked without providing the possible responses so that we can see if water quality is mentioned as a problem without prompting.
4	Have you noticed a change in the quality of your water in the last two weeks?	Yes/No	
4a	**IF** yes, please identify which source had the changed water quality? [*If multiple sources changed, ask them about the most important change*]	[text field]	We need to know the source to link it to observations from the diaries, otherwise it would be more difficult to attribute a changed behaviour to an observed change in water quality.
4b	**IF** yes, please describe the water quality change?	[text field]	
4c	**IF** yes, do you have concerns about the change?	Yes/No	
4c	**IF** yes, what are you concerned about?	[text field]	
4d	**IF** no, why are you not concerned?	[text field]	Capture the non-concern rationale and avoid temptation of answering 'no concern' to just shorten the survey.

Supplementary File S1: Longitudinal Household Survey Check-in Questionnaire

Section 2: Health Questions

	**Question**	**Response**	**Notes**
1	How many people lived in your household in the last 2 weeks?	[numeric]	
2	How many people in your household have been ill in the last two weeks?	[numeric] -> will generate looping	Looped in ONA to capture if multiple members have illnesses.
2a	**IF** yes, what is their age?	a) Young child (less than 5)<br>b) Child (5 to 14)<br>c) Young Adult (15 to 20)<br>d) Adult (20 to 60)<br>e) Senior (over 60)	
2b	**IF** yes, what are the symptoms of the illness?<br>[*select all that apply*]	a) Diarrhoea<br>b) Cough<br>c) Skin infection<br>d) Headache<br>e) Stomach cramps<br>f) Fever<br>g) Other [text field]	
2c	**IF** yes, do you know the reason why they are sick?<br>[*do not prompt, if they don't know a or do not respond, just say 'DK' or 'NR'*]	[text field]	We can trial this question with instructions not to prompt possible responses. We can remove the question if it is not working based on responses coming in and feedback from enumerators.
2d	**IF** yes, have they had medicine and / or visited a hospital or clinic for treatment?<br>All that apply	a) No<br>b) Yes – traditional medicine<br>c) Yes – prescribed medicine<br>d) Yes - they have visited a hospital or clinic	Interesting to see if those who are proactive in terms of seeking medical care or medicine are also proactive in dealing with water quality concerns.

Supplementary File S2: Water Quality Sampling and Analysis Protocol

Acronyms and Abbreviations

E. coli – Escherichia coli
CDOM – coloured dissolved organic matter
MPN – most probable number
TLF – tryptophan-like fluorescence
WP – water point
WQ – water quality

Contents

Equipment 2
Schedule 2
Field Instructions 3
Equipment and supplies to bring: 3
Recording results and observations: 4
Notes for good fieldwork: 4
Taking in-situ measurements: 5
Turbidity 5
Conductivity, Salinity and Temperature 6
TLF and CDOM 7
Collecting Samples for lab analysis: 9
Other considerations while in the field: 9
Returning from the field: 10
Lab Instructions 10
E. coli Sample Processing Procedure 11
Most Probably Number Procedure 12
pH and fluoride 12
Daily Care of Equipment 14
Quality Assurance 15
Duplicates – E. coli and fluoride 15
Blanks – E. coli, fluoride and TLF 15
Calibration Checks 16
Recalibration 16
Turbidity 16
pH 17
Fluoride 17
Conductivity 18

Equipment

The following instruments are used in this protocol. Detailed application notes and user manuals are available online for each of them.

Equipment	ID	Documentation References
Hach multimeter	HQ 40D	User Manual DOC022.98.80017
Hach conductivity probe	CDC40101	User Manual DOC022.52.80022
Hach pH probe	PHC10101	User Manual DOC022.52.80023
Hach fluoride probe	ISEF12101	User Manual DOC022.53.80028
Hanna turbidimeter	HI93703	Instruction Manual HI93703
IDEXX Quanti-tray system	n/a	Quanti-Tray/2000 User Instructions IDEXX 06-02320-14<br>Spectronics E-Series UV Hand Lamps User Manual 86131-12<br>Colilert-18 Procedure IDEXX 06-02027-24
Boekel TTT Incubator	135000	Operating Instructions N2400342
Chelsea Technologies Group UviLux system	n/a	Hawk Data and Logging Unit Product Specifications<br>CTG UviLux Datasheet<br>Ward, J. *et al.* (2018) *Assessing microbiological contamination in groundwater sources: Field note on using Tryptophan-like Fluorescence (TLF) Probes. British Geological Survey Open Report, OR/18/042.*

Schedule

The following tables outline the daily schedules to be followed each week with site visits in the morning to early afternoon and lab work in the late afternoon.

Daily Activities for Monday to Friday	Tasks
Visiting sites.	Conduct in-situ tests, collect samples for *E. coli* analysis, and maintain good relationships with committee members, managers and users.
Return to lab.	Finish site visits and return to the lab by 15:00 to 15:30.
Process *E. coli* samples.	Follow the lab protocol for *E. coli* analysis to put samples into the incubator.
Confirm *E. coli* results from the previous day.	Check and record the results of the *E. coli* samples from the previous day following the *E. coli* results protocol.
Process fluoride samples.	Acidify and measure fluoride for all samples following protocol A or B as appropriate.
Prepare for next day.	Clean and store the sampling equipment in preparation for the next day following the appropriate protocols. Confirm sampling plan for the next day.
Record field results and observations.	Enter the test results and observations and comments from the field into the WQ_Monitoring_WeeklyReport_##.
Clarifications and modifications.	Respond to data report comments and questions as needed.

Saturday Activities	Tasks
Do TLF blank sample.	Follow the normal TLF sampling process using fresh bottled water as the sample.

Check calibrations.	Check the calibrations of the turbidity, pH and conductivity probes and recalibrate if necessary.
Confirm *E. coli* results from the previous day.	Check and record the results of the *E. coli* samples from the previous day.
Finalise the weekly report.	Finalise the WQ_Monitoring_WeeklyReport_wk#.
Cleaning.	Thoroughly clean the lab, clear out any garbage and wash the kikois and small hand towels.

Field Instructions

Equipment and supplies to bring:

Before leaving the lab for the day, make sure you have the following items:

1. In backpack:
    a. Wristwatch or other device to record time.
    b. Permanent marker for labelling sample bottles.
    c. Pen and notebook for recording results and observations.
    d. Hand sanitizer.
    e. Bottle of ethanol.
    f. Cotton wool.
    g. Metal tongs.
    h. Lighter or matches.
    i. Enough sample bottles for taking *E. coli* samples.
    j. Enough sample bottles for taking fluoride samples.
    k. Hawk meter for **TLF** and **CDOM** measurements.
    l. Hach multimeter with **conductivity** probe.
    m. Plastic beaker for conductivity measurement.
    n. Hanna **turbidity** meter and glass cuvette.
    o. Small cloth for drying probes and cuvette.
    p. Soft, clean cloth for polishing turbidity cuvette to remove all smudges.
    q. GPS device (optional).
2. Plastic basin with bubble wrap and cloth for transporting TLF and CDOM equipment.
3. **TLF** and **CDOM** sensors with cables.
4. Plastic bucket with lid and metal pot for TLF and CDOM tests.
5. Metal bucket and rope if you will be sampling any reservoirs or open wells.
6. Cooler box with ice-packs and a thermometer for transporting *E. coli* samples.
7. Bottled water for rinsing equipment between sites.
8. Container for garbage.

Recording results and observations:

For each sampling visit to a site, you need to record the following information. Make sure that you also leave space in your notebook to record the **pH** and **fluoride** results when you do the measurements in the lab at the end of the day.

Who is doing the sampling:	e.g. Musenya and Mbogo
Site ID:	e.g. KK-30
Start time:	e.g. 13:05
Weather:	e.g. No rain today, very hot, clear skies
Order of visit today:	e.g. 3rd
Were people observed using the WP?	e.g. yes, it was busy there was a line
Date of last pumping:	e.g. 26-Nov-2018
Date of last breakdown:	e.g. 15-Dec-2018
**Turbidity (FTU):**	e.g. 1.06
**Colour:**	e.g. faint yellow-green
**Temperature (°C)**:	e.g. 27
**Conductivity (µS/cm)**:	e.g. 987
**Salinity (ppt):**	e.g.
**TLF (ppm)** (record 6 observations):	e.g. 1.71, 1.71, 1.73, 1.69, 1.70, 1.71
**CDOM (ppm)** (record 6 observations):	e.g. 4.52, 4.55, 4.56, 4.55, 4.49, 4.51
Did you use fire to disinfect the spout or tap?	e.g. yes or not applicable
Did you collect an ***E. coli*** sample?	e.g. yes or no
Did you collect a **duplicate** *E. coli* sample?	e.g. yes or no
Did you collect a **fluoride** sample?	e.g. yes or no
Did you collect a **duplicate** fluoride sample?	e.g. yes or no
Your observations (what you noticed while at the site):	e.g. The WP was very busy, we had to wait to take a sample. The pH probe took a while to stabilise. There were many goats and donkeys around.
Comments from committee, managers or users:	e.g. A man told us that the water is tasting better now that it has been raining for some time. A committee member was asking us when we will bring results. A woman told us that the water is giving her stomach problems.
End time:	e.g. 13:25

Notes for good fieldwork:

Before leaving Kyuso, make sure you know your route for the day and who you will need to contact to unlock sources or turn on pumps etc. to give you access for sampling. Let Peter or somebody else at the office know where you plan to be working. Make sure you have water and food to keep hydrated and have good energy for the day. Bring phones with enough battery life and airtime to call for assistance if you need it. For the sites that you can access by car, work out of the back of the car so you can keep the equipment clean and out of the direct sun as much as possible. For sites that require walking, leave the cooler box in the car and bring the other equipment that you need. Please check that the car is secure and locked before you leave it. When working with the equipment outside of the car – keep it as clean as possible (do not let cables drag on the ground). Keep equipment in the shade when possible so that it doesn't overheat and so the sun does not interfere with measurements. You should aim to return to the lab by 15:00 or 15:30, so plan your day

accordingly. Your safety comes first. ff you cannot reach some sites because of road conditions or other reasons, make a note of why you could not go.

Taking in-situ measurements:

Follow the protocols below to take measurements for turbidity, temperature, conductivity, salinity, TLF and CDOM. If you are sampling from a borehole, allow the water to flow for a few minutes to flush the borehole before taking samples. Remember to take measurements of samples as soon as you can after collecting them. Do not allow samples to sit in the containers for some time before measuring because the parameters (e.g. temperature) will change.

Turbidity

1. Rinse the glass cuvette three times with sample water.
2. Dry the cuvette and allow some time for bubbles to escape before putting the cap on.
3. Put the cap on – try to always tighten it the same amount.
4. Secure the cap and wipe the cuvette with a clean, dry, soft cloth to remove any smudges.
5. Hold the cuvette up to the light to check that it is dry and clean. The cuvette must be completely free of fingerprints and other oil or dirt, particularly in the area where the light goes through (approximately the bottom 2 cm/1 inch of the cuvette).
6. Turn the meter on by pressing the ON/OFF key.
7. When the display screen shows "----" the meter is ready to measure.
8. Gently rotate the cuvette so that the sample is mixed without creating any bubbles.
9. Place the cuvette in the opening and check that the notch on the cap is positioned correctly into the groove. The mark on the cuvette cap should be facing toward the display screen.
10. Place the meter on a flat surface.
11. Press the READ key, the LCD will display a blinking "SIP" (Sampling in Process). Do not move the meter while sampling is in process.
12. After about 25 seconds the result will appear on the display screen.
13. Remove the cuvette and rotate it slowly to mix the sample without adding bubbles. Check again that the glass is clear. Put the cuvette back in the opening and press the READ key again. Continue until you have some consistent results. *Note: results of 40 FTU or greater will not be very accurate, so it is okay if you get some variable measurements above 40 FTU - you can take the average of the readings.*
14. Discard the sample after you have finished recording the reading.
15. Rinse the cuvette with bottled water.
16. Wrap it with tissue and put it back in the small plastic bag.
17. Put the cap back on the opening of the meter.
18. Put the meter and cuvette back in the backpack.

***Note that to maximize the battery life the meter is automatically switched off after 5 minutes of non-use. To reactivate it, simply press the ON/OFF key.

Conductivity, Salinity and Temperature

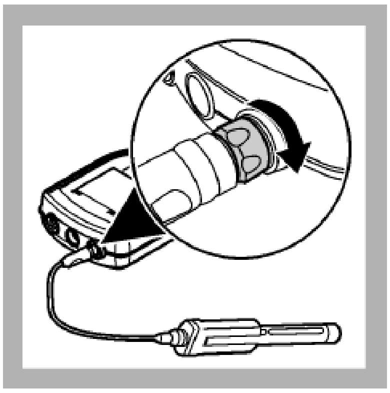

1. Rinse the plastic beaker three times with the sample water.
2. Rinse the probe with the sample water.
3. Fill the plastic beaker three quarters full of sample water.
4. Check that the probe is properly connected to the multimeter. Make sure the cable locking nut is securely tightened.
5. Turn on the meter.
6. Place the probes in the sample. Make sure that measurement areas of the probe is completely submerged.

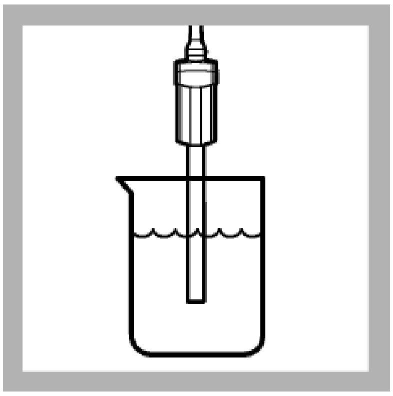

7. Move the probe gently in the sample and ensure that the measurement areas remain submerged for the entire time that the sample is being read. Do not put the probes against the bottom or sides of the container. Note that air bubbles on the sensors can cause slow stabilizations or measurement errors, gently moving the probe will help to remove bubbles.
8. Press the 'Read' button. The display will show 'Stabilizing' and a progress bar. When the reading is stable, the progress bar will disappear, and a lock icon will be shown.
9. Record the stabilised measurements including the temperature, conductivity, and salinity.
10. Turn off the meter.
11. Rinse the probe with bottled water then blot dry them with a clean cloth.
12. Wrap the probe in something soft to protect it during transport.
13. Place the multimeter and probe into the backpack. Make sure that the cables are not bent too much so they don't get damaged.

Supplementary File S2: Water Quality Sampling and Analysis Protocol

TLF and CDOM

1. Put the plastic bucket in a shaded area if possible, to minimise interference from the sun.
2. Clean the sample container (metal pot) with ethanol if it is dirty. Rinse it at least three times with sample water. Use the rinse water to splash onto the TLF and CDOM probes.
3. Rinse the TLF and CDOM probes directly under the sample water if possible (or splash it with water drawn from reservoir, scooping pit or shallow well – you can use the metal bucket for this but make sure it is clean and rinsed first).
4. Fill the sample container (metal pot) halfway, be very careful not to introduce contamination to the sample. Don't touch the inside of the container, avoid letting any dust get into the container and don't let water run down from your hands into the container.
5. Place the container with the sample in it inside the plastic bucket.
6. Place the probes into the sample. Move them gently to make sure no air bubbles are trapped inside the sensor window.
7. Take care to avoid letting the cables touch the sample water.
8. Place a cover over the plastic bucket.
9. Connect the Hawk meter to the cable and then turn it on.
10. Record 6 TLF measurements by having one person glancing back and forth to the display and reading the value out loud for the second person to write down.
11. Repeat this process to record 6 CDOM measurements.
12. Observe if the 6 readings are consistent or if there is an increasing or decreasing trend. If you see a trend in the 6 measurements this could be due to:
    a. Suspended particles settling out of the sample. If you suspect this is the problem. Allow the probe to rest in the sample for a few minutes before taking 6 readings again.
    b. There is contamination on the probe or the sampling container that is causing the value to increase. If you suspect this is the problem. Repeat steps 2 to 11 and take extra care with how you clean the sampling container and probe to avoid introducing contamination.
13. Remove the probes from the sample, take care not to touch them with the cables or to any other surface.
14. Dispose of the sample without touching the inside of the sample container.
15. Repeat steps 2 to 13 for a second sample.
16. Check if the results for the second sample are similar to the first.
    - If they are similar, you can move on to step 16.
    - If the second sample is less than or higher than the first sample, repeat steps 2 to 13 for a third sample.
        - If the third sample is in-between the first and second samples, you can move on to step 16.
        - If the third sample shows the measurements are still getting smaller or still getting larger, continue with steps 2 to 13 for more samples until the results are consistent.

17. Turn off the Hawk meter, then disconnect it from the cables.
18. Rinse the probes with bottled water and place them carefully in the transport basin. Cover them with the cloth and place the cables around them. Take care that none of the cables are too bent and that they are kept clean.
19. Rinse the sample container with bottled water. Place it inside the plastic bucket with the lid on for transport.
20. Place the Hawk meter back in the backpack. Make sure it is turned off otherwise the battery will drain.

Note: negative readings (close to zero) are possible for TLF if the water is very, very clean. However, negative reading can also be caused by bubbles in the sensor window so make sure to gently move the probe to remove any bubbles.

The main issues to be aware of when taking TLF measurements are:

i. **Air bubbles** can get trapped in the sensor window where the observations are made, these need to be removed before taking readings otherwise the readings will be too low or negative. Bubble effects can be minimised by placing the sensor in the container at an angle to minimise the likelihood of air being trapped and by swirling the sensor in the container to remove any small air bubbles that are on the sensor.

ii. **Secondary contamination of samples** must be avoided. The sample can be contaminated by TLF material coming off of contaminated containers and/or contaminated probes. This kind of contamination can happen if the probes are not handled or stored properly. Secondary contamination is minimised by doing the following:

    a. Rinse the probes and sampling container with clean water after each sample and clean them thoroughly with ethanol in the lab every evening.

    b. Make sure the equipment, including the cables, is not put on the ground or other dirty surfaces. They should be kept in clean containers (the transport basin, lab basin, and plastic bucket).

    c. Use gloves whenever touching the part of the probes that goes in the sample. This includes when you are cleaning the probes in the evening.

    d. Doing blank samples – this means using bottled water as the sample – to confirm that secondary contamination is minimal.

iii. **UV light** (sunshine) will affect reading level and stability. This can be avoided by keeping the lid on the bucket when taking measurements and doing the measurement in the shade whenever possible.

iv. **High turbidity** may influence TLF readings. If the water has a lot of suspended particles in it. Allow time for the particles to settle out before taking the TLF measurement.

v. **Temperature changes and pH and conductivity** may also influence TLF measurements. To deal with this, it is important to always record the temperature, pH and conductivity when you are sampling so that a correction calculation can be applied to the TLF measurement if necessary.

vi. **Equipment connections**: the cable connections between the probes and the Hawk meter are weak points. These can be easily damaged if they are bent too strongly or if there is too much pressure pulling on them. This should be avoided by holding the equipment only from the strong areas of the cables and the metal rings, and by storing and transporting the equipment so that the cables are not strongly bent.

Supplementary File S2: Water Quality Sampling and Analysis Protocol

Collecting Samples for lab analysis:

Samples for *E. coli* Analysis:

1. If the source has a metal spout or tap with no plastic, use the tongs, cotton wool, ethanol and lighter to disinfect the spout for about 1 – 2 minutes. Be careful to fully extinguish the cotton wool when you are finished and put it in a garbage container.
2. Wait for a few minutes for the disinfected area to cool down. During this time, make sure nobody uses or touches the spout.
3. Put hand sanitizer on your hands before holding the sample bottle.
4. Label the sample bottle with the Site ID and the date.
5. Run the water through the spout for approximately 20 L.
6. Open the sample bottle being careful to avoid touching the opening or the inside of the cap.
7. Fill the container directly from the spout where possible or by pouring from a cleaned and rinsed container.
8. The volume of the sample should be **100 mL or a little bit more than 100 mL**. If you have too much, pour a bit out and refill as necessary to achieve close to 100 mL line. This is important to avoid spilling during sample processing later.
9. When you are happy with the sample volume, close the container tightly.
10. As soon as possible, place the container inside the cooler box. Take care to open and close the cooler box as quickly as possible to avoid letting is warm up too much.

Samples for pH and fluoride analysis:

1. Open the amber glass bottle and rinse it at least three times with the sample water – including rinsing the lid.
2. Fill the bottle with sample water leaving a few centimetres of air space at the top.
3. Record the number on the sample bottle in your notebook so that you are certain which bottle corresponds to the site.
4. Store the bottle securely in a bag and keep it out of the sun.

Other considerations while in the field:

While you are busy with the sampling, you must remember to do the following:

- **Call the office** to make sure that somebody will take the *E. coli* samples out of the incubator within the appropriate 4-hour time period and that they will record the results.
- **Plan your route for tomorrow** and make calls to ensure that you will have access to the sites that you need e.g. somebody to unlock or turn on a pump as necessary.
    - Remember that only 12 *E. coli* trays can be incubated at one time, so plan to collect no more than 12 *E. coli* samples on any one day.
- Don't forget to stay hydrated and have some food!

Returning from the field:

When you return to the office, remember to:

- Unload the equipment.
- Check if you need to buy more bottled water for tomorrow.
- Check that the car is in good condition – including removing garbage, cleaning dirt from the back if necessary, checking that the tires are okay and checking if refuelling is needed before tomorrow.
- Record the mileage from the day in the log book.

Lab Instructions

Take the following steps when you return to the lab in the afternoon:

1. Record the temperature from the thermometer in the cooler box.
2. Put the *E. coli* and fluoride samples on the table so they can come to room temperature.
3. Put the ice packs in the freezer.
4. Process the *E. coli* samples according to the instructions below.
5. Double-check the results for the *E. coli* samples from the previous day.
6. Put the *E. coli* waste in the dedicated ‘biowaste’ garbage drum.
7. Sanitize the tables by wiping them with ethanol.
8. Process the pH / fluoride samples according to the instructions below.
9. Clean all the field equipment and make it ready for tomorrow.
10. Confirm sampling plan for tomorrow and how many *E. coli* sample bottles are needed.
11. Record results in the WQ_Monitoring_WeeklyReport_wk# (This must be completed and shared for review every Saturday).
12. Before leaving the lab make sure that:
    a. Everything is in the correct location and there is no clutter on the counters or the floors.
    b. The incubator window is covered.
    c. The quantitray sealer and UV lamp are turned off.
    d. The fridge door is firmly closed.
    e. The lights are off.
    f. The curtains are drawn.
    g. Both doors are securely locked.

E. coli Sample Processing Procedure

1. Allow samples to warm to room temperature.
2. Turn on the quantitray sealer so it can warm up – it is ready when the light goes green.
3. Turn on incubator so it can warm up to 35°C.
4. Tear off the blister pack of Colilert-18 media. Take the number that you need and put the rest back in the fridge.

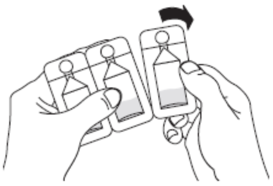

5. Open a sample bottle.
6. Tap a blister pack gently. Turn the pack so that it is facing the sample bottle. Snap back the lid to open the pack and pour the Colilert into the sample. Gently tap to get all of the powder into the sample.

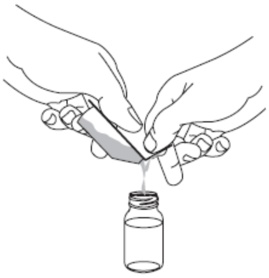

7. Close the sample bottle.
8. Repeat steps 5 to 7 for all of the samples.
9. Gently rotate the sample bottles to help the Colilert dissolve but be careful not to create bubbles.
10. Once dissolved, put the samples back on the table and open them to allow the bubbles to disappear.
11. Take a quantitray and squeeze it near the top with one hand to open it. The wells should be facing your palm. Gently pull the metallic tab backward to make the opening bigger if you need to but **be careful not to touch inside the tray**.

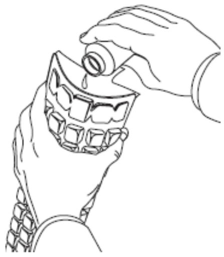

12. Pour a sample into the tray. Try to pour at a moderate speed so that you don't introduce bubbles and so that no spilling occurs.
13. Tilt the tray slightly so that you can gently tap it to release any bubbles. Hold the tray for a minute or two to allow foam/bubbles to dissipate.
14. Keep the tray at a slight angle to prevent spilling and place it inside the rubber insert.
15. Place the rubber insert at the entrance of the Quantitray sealer and make sure it is straight.
16. Gently push the tray into the sealer until it catches and is pulled through. If the tray is being pulled through at a crooked angle, you can press the reverse button immediately to push it out and straighten it.

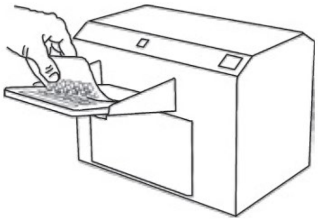

17. Wait until the sealer stops making a noise before you pull the tray out on the other side.
18. Write the Site ID and date on the back of the tray. If it is a duplicate sample, write DUP.
19. Repeat steps 11 to 18 for all of the samples.
20. Put the samples in the incubator and cover the window with a small cloth.
21. Fill out the incubation record form with the time that you put the trays into the incubator and calculate the minimum (+18 hours) and maximum (+22 hours) completion time for incubation.
22. After double-checking the results of the samples from the previous day according to the most probably number procedure. Clean the tables with ethanol.

Most Probably Number Procedure

1. Wear gloves when handling E. coli trays.
2. Record the Site ID, date, and whether it is a duplicate sample of not.
3. Count and record the number of large yellow wells.
4. Count and record the number of small yellow wells.
5. Count and record the number of large wells that are yellow and glowing under the UV light.
6. Count and record the number of small wells that are yellow and glowing under the UV light.
7. Mark the glowing wells with a black dot from the permanent marker. If most of the wells are glowing, you can circle the ones that are **not** glowing as an alternative to marking dots on most of the wells.
8. Look up the results on the most probable number (MPN) table.

pH and fluoride

1. Put gloves on.
2. If any of the samples are expected to be less than 1 mg/L of fluoride,
    a. rinse the 100mL plastic beaker three times with deionised water;
    b. pour 50mL of deionised water into the beaker;
    c. empty the contents of one buffer capsule into the deionised water and use a clean pastette (either freshly unwrapped or cleaned with deionised water) to stir the solution until the buffer acid dissolves.
3. Connect the pH and fluoride probes to the multimeter. Make sure the cable locking nuts are securely tightened. Remove the protective caps from the probes. Turn on the meter.
4. Prepare your sample:
    a. Rinse the plastic 50mL beaker with deionised water and then three times with sample water.
    b. Pour 25mL of sample water into the beaker.

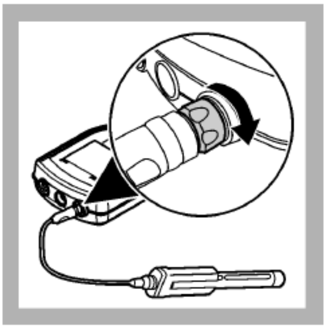

5. Rinse the pH probe with deionised water and gently shake it to remove excess water drops.
6. Place the pH probe in the sample. Make sure that measurement areas of the probe are completely submerged.
7. Move the pH probe gently in the sample and ensure that the measurement areas remain submerged for the entire time that the sample is being read. Do not put the probe against the bottom or sides of the container. Note that air bubbles on the sensor can cause slow stabilizations or measurement errors.
8. Press the ‘Read’ button. The display will show ‘Stabilizing’ and a progress bar. When the reading is stable, the progress bar will disappear, and a lock icon will be shown.

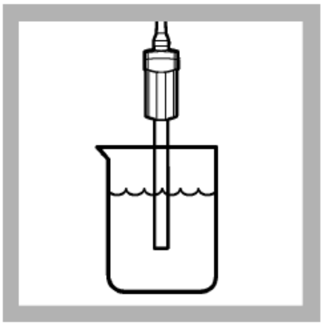

9. Record the stabilised pH measurement and remove the pH probe from the sample.
10. Acidify the sample:
    a. If the sample is expected to have fluoride less than (<) 1 mg/L, follow **protocol A** for acidification:
        i. Using a clean pastette, transfer 5mL of dilute buffer solution (prepared in step 1) into the sample. Be careful not to touch the pastette to the sample water.
    b. If the sample is expected to have fluoride greater than (>) 1 mg/L, follow **protocol B** for acidification:
        i. Empty the contents of a buffer sachet into the sample and use a clean pastette to stir the sample until the buffer powder is dissolved. The pastette must be freshly unwrapped or rinsed with deionised water before it is put into the sample. To avoid cross contamination, the pastette should not be reused for other samples.
    c. If this is the first fluoride sample for a site and you are not sure what concentration to expect, start with protocol A. If the result from protocol A is >1mg/L, repeat the analysis again using protocol B.
11. Rinse the fluoride probe with deionised water before placing it in the sample.
12. Move the probe gently in the sample and ensure that the measurement areas remain submerged for the entire time that the sample is being read. Do not put the probe against the bottom or sides of the container. Note that air bubbles on the sensor can cause slow stabilizations or measurement errors.
13. Press the 'Read' button. The display will show 'Stabilizing' and a progress bar. When the reading is stable, the progress bar will disappear, and a lock icon will be shown.
14. Record the stabilised fluoride measurement <u>and temperature</u> and remove the probe from the sample.
15. Repeat steps 4-14 for all of the samples.
16. Turn off the meter and disconnect the probes.
17. Place the storage caps on both probes. The fluoride probe can be stored dry. For the pH probe, make sure there is some pH storage solution in the probe cap. **The bulb and reference junctions of the pH probe should not be allowed to dry out.** Make sure there is enough storage solution in the cap to completely cover the blub and reference junctions.
18. Return the probes to the shelf. Rinse the amber glass sample bottles with bottled water before closing them and returning them to the shelf. Return the buffer capsules to the fridge and rinse the plastic beakers with deionised water before returning them to the shelf.

Supplementary File S2: Water Quality Sampling and Analysis Protocol

Daily Care of Equipment

Before closing the lab for the day, please complete the following tasks:

1. Clean the tables with ethanol and turn off the sealer and UV light.
2. Clean the equipment:
    a. Rinse the turbidity cuvette with deionized water.
    b. Rinse the conductivity probe with deionized water and blot dry it.
    c. Rinse the pH probe with deionized water and check that there is sufficient storage solution in the storage cap. There must be enough liquid to cover the bulb of the probe when the cap is on. Replace the solution if necessary. The glass bulb must not be allowed to dry out. If it becomes dry:
        i. Soak the probe tip in the 4.01, 7.00 and 10.01 buffers for 5 minutes each.
        ii. Rinse the probe with deionized water. Blot dry with a lint-free cloth.
        iii. Calibrate the probe.
    d. Clean the TLF and CDOM probes and sampling container with ethanol and transfer them to the clean lab basin for storage overnight. Use gloves while handling the probes. Check that the cables are clean of any dirt. Let the plastic bucket dry out.
3. Make sure that the inside of the backpack is clean and dry.
4. Make sure that the wiping cloths for the field are clean and dry. If not, replace them with a clean set and plan to get the dirty ones washed.
5. Check that the multimeter, turbidity meter, and Hawk meter are clean and dry. If they are dirty, clean with some water and a soft cloth and / or Q-tips.
6. Check you have enough cotton wool, lighter or matches, ethanol, and hand sanitizer in the backpack for sampling tomorrow.
7. Plug the Hawk meter into the charger.
8. Replace the batteries on the turbidity meter and multimeter if they are indicating low battery warnings. Both meters use AA alkaline batteries.

For the turbidity meter:

a. A "**LO BAT**" indication will appear on the lower right corner of the display when the batteries are weak and require replacement. The instrument can still perform approx. 50 measurements. A "**-BA-**" indication will appear on the display when the batteries are too weak to perform reliable measurements. The message appears for a few seconds, and then the meter will automatically switch off.
b. To install or replace the batteries, turn the unit off and unscrew the 2 screws located on the back of the meter. Remove the battery cover and insert the new batteries in the compartment while paying attention to the polarity. After the batteries have been installed, close the battery cover and tighten the 2 screws.

For the multimeter:

c. The battery symbol on the display will indicate when the batteries are low.
d. To replace them, pull the release tab on the battery cover and insert the new batteries paying attention to the polarity. Then slide the cover back in place.
e. Note that the batter compartment is not waterproof. If the compartment becomes wet, remove and dry the batteries and dry the interior of the compartment. Check the battery contacts for corrosion before replacing them.

Quality Assurance

Duplicates – E. coli and fluoride

You must collect duplicate samples to check the accuracy of the methods. A duplicate sample is a second sample collected directly after the first one in the same way. *E. coli* duplicates should be labelled with the Site ID, date, and the letters 'DUP'. Fluoride duplicates should be identified in the notebook using the number on the amber glass bottle.

Collect one duplicate sample for *E. coli* and fluoride every week. The site for the duplicate should be randomly selected each week.

Blanks – E. coli, fluoride and TLF

To check that secondary contamination is not impacting your samples. It will be important to do blank samples for *E. coli*, fluoride, and TLF. Blank samples are when you use botted or deionised water as the sample water. You should do at least one blank sample every week for *E. coli*, fluoride, and TLF:

E. coli

For *E. coli*, collect the blank sample using the same procedure that you use for normal samples. Do this while you are out in the field at a site. Use hand sanitizer before you start. Use a fresh bottled water – not one that has already been open for some time. Pour the bottled water into the *E. coli* sample container up to the 100 mL line. Close the sample tightly and place it in the cooler box with the other *E. coli* samples from that day. This sample should be labelled with 'BLK' and the date. It should be processed in the same way as the other samples.

You should do a blank sample for *E. coli* once per week. You can choose which day you do this. Remember that only 12 trays can fit in the incubator at any time, so choose to do a blank on a day when you have only 11 or less *E. coli* samples to process.

Fluoride

For fluoride, half-fill a randomly chosen amber glass bottle with deionised water in the morning. Carry this bottle with you during the fieldwork for the day and analyse it along with the other fluoride samples in the evening.

TLF

For TLF, blanks will also be conducted using bottled water. Again, this should be fresh bottled water not a bottle that has been open for some time. This can be done at the office and you should follow the same procedure that you would use at a site – including rinsing the sampling container and probes very well with the bottled water before taking a measurement.

The TLF blanks can be done on Saturday mornings.

Calibration Checks

Check the accuracy of the turbidity, pH, fluoride, and conductivity probes every Saturday using the standard solutions. If the values are not within 5% of the standard value, recalibrate the probe.

Parameter	Standard	Minimum OK Reading	Maximum OK Reading
Turbidity (FTU)	0<br>10<br>500	0<br>9<br>450	0<br>11<br>550
pH	4<br>7<br>10	3.8<br>6.65<br>9.5	4.2<br>7.35<br>10.5
fluoride	0.5<br>1<br>2	After I see the weekly results report, I will let you know if the probe need recalibration. If you notice the reading is far off from the standard, please point it out in your email.	
Conductivity (µS/cm)	1413	1342 @25°C<br>The correct reading depends on the temperature. Check the calibration standard to match the temperature to the correct concentration. The probe is okay if it reads within ±100µS/cm	1483 @25°C

Recalibration

If the calibration checks fail, follow these protocols to recalibrate.

Note: make sure you have enough standard solution remaining to complete the calibration process before you start!

Turbidity

1. Rinse the turbidity cuvette thoroughly with deionized water to prepare for calibration.
2. Turn the meter on and wait for the display to show "----".
3. Press the CAL key once, the "CAL" message will blink on the display for about 6 seconds, then the calibration mode stops.
4. While the "CAL" message is still blinking, press CAL again. The instrument is now in the calibration mode and a "CL" will appear on the lower part of the display.
5. To confirm the displayed date values and to go to the next step, press the CAL key once. A blinking "ZERO" message will appear.
6. Take the HI 93703-0 bottle containing the ZERO FTU standard and fill the measurement cuvette. Fill slowly and pour the liquid down the side of the cuvette if possible, to reduce air bubbles. Dry and polish the glass so there are no marks.
7. Insert the cuvette with the ZERO FTU standard solution into the measurement cell and press the CAL key. A blinking "SIP" message indicates that the instrument is performing the measurement.
8. After approximately 30 seconds the instrument will ask for the HI 93703-10 standard solution of 10 FTU by displaying "10.0".
9. Repeat steps 5 and 6 with the 10 FTU standard solution. After the second calibration point (10.00 FTU) has been accepted, the meter will display “500”, asking for the 500 FTU solution to be placed in the cuvette holder. *Note: At this point the user can exit the calibration mode and save the two-point calibration by pressing READ.*

10. To perform a three-point calibration, repeat steps 5 and 6 with the 500 FTU standard solution.
11. After approximately 30 seconds, the calibration process will be complete, and the display will show "----". Now the meter is calibrated and ready for use.

Note*: In order to minimize any error introduced by the cuvette, it is recommended to use, during calibration, the same cuvette you are going to use to perform the measurement.*

pH

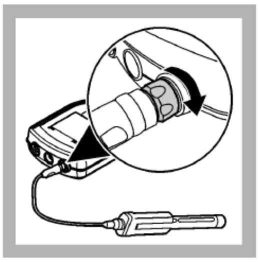

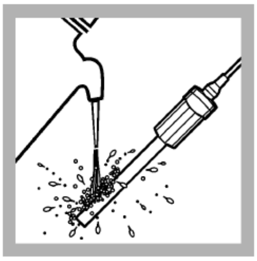

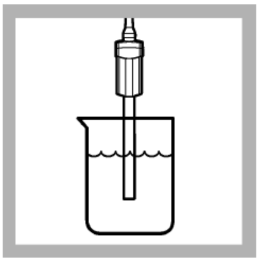

1. Connect the probe to the meter. Make sure that the cable locking nut is securely connected to the meter. Turn on the meter.
2. If multiple probes are connected, push the **UP** or **DOWN** arrow to change to the single display mode in order to show the calibrate option.
3. Push **Calibrate**. The display shows the necessary buffers.
4. Prepare the fresh buffers in the plastic beakers that are labelled 4, 7, and 10.
5. Rinse the probe with deionized water and blot it dry with a clean cloth.
6. Put the probe in the pH 4.01 buffer solution and stir gently. Make sure that the reference junctions are completely submerged.
7. Push **Read** and stir the probe gently in the solution. The display will show "Stabilizing" and a progress bar as the probe stabilizes in the standard.
8. When stabilized, the display shows the buffer that has just been read and shows the temperature corrected pH value.
9. Repeat steps 5 to 8 with pH buffer solutions of 7 and 10.
10. Push **Done** to view the calibration summary and record the slope, offset and r^2 values. *Note: The display will not show Done until all three calibration points have been collected.*
11. Push **Store** to accept the calibration and go back to measurement mode.

To change calibration options:
Please do not change the calibration options. If you have a concern, please let me know.

Fluoride

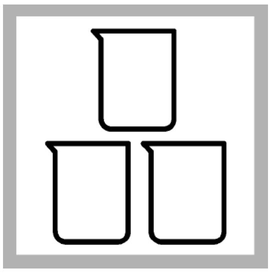

1. Take the standard solutions out of the fridge and allow them to warm to room temperature.
2. Put gloves on.
3. In three separate beakers (use the two 50mL and one of the 100mL plastic beakers) prepare the three standard solutions – 0.5 mg/L, 1 mg/L and 2 mg/L:
    a. Rinse the beakers with three times with deionised water and once with the standard solution.
    b. Pour 25mL of the standard solution into the beaker.
    c. Add one buffer capsule to each beaker and use clean pastettes to stir until the buffer is dissolved in each standard.
4. Connect the probes to the meter. Make sure that the cable locking nut is securely connected to the meter. Remove the protective caps from the probes. Turn on the meter.

5. If multiple probes are connected, push the **UP** or **DOWN** arrow to change to the single display mode in order to show the calibrate option.

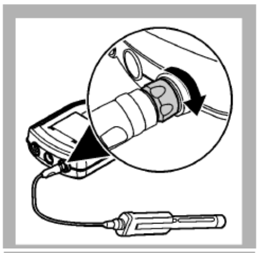

6. Rinse the probe with deionized water and gently shake it to remove excess droplets.
7. Push **Calibrate**. The display shows the buffers that are necessary for calibration.
8. Put the probe in the 0.5 mg/L standard and stir gently. Make sure that the reference junctions are completely submerged.

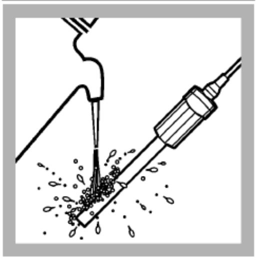

9. Push **Read** and stir the probe gently in the solution. Make sure there are no bubbles on the probe and do not touch the bottom or sides of the beaker. The display will show "Stabilizing" and a progress bar as the probe stabilizes in the standard.
10. When the value has stabilised, the display will ask for the 1 mg/L standard.
11. DO NOT RINSE THE PROBE. Shake it gently and place it directly into the 1mg/L standard.

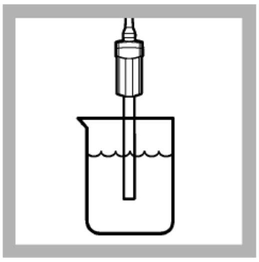

12. The display will then ask for the 2mg/L standard. Again, do not rinse the probe but shake it gently and then place it into the 2 mg/L standard.
    Push **Done** to view the calibration summary and record the results for the weekly results report. Make sure you include the temperature of each of the standards!
    Note: The display will not show Done until all three calibration points have been collected.
13. Push **Store** to accept the calibration and go back to measurement mode.

Conductivity

1. Connect the probe to the meter. Make sure that the cable locking nut is securely connected to the meter. Turn on the meter.
2. If multiple probes are connected, push the **UP** or **DOWN** arrow to change to the single display mode in order to show the calibrate option.
3. Push **Calibrate**. The display shows the conductivity standard solution that is being requested for calibration. *Note: You can change which conductivity standard is being requested in the Calibration Options menu.*
4. Add fresh conductivity standard solution to the plastic beaker that is marked for conductivity standard.
5. Rinse the probe with deionized water and blot it dry with a clean cloth.
6. Put the probe in the standard solution and stir gently. Make sure that the temperature sensor is completely submerged.
7. Push **Read** and stir the probe gently in the solution. The display will show "Stabilizing" and a progress bar as the probe stabilizes in the standard.
8. When stabilised, the display shows the standard solution value that has just been read and shows the temperature corrected value.
9. Push **Done** to view the calibration summary.
10. Push **Store** to accept the calibration and return to the measurement mode.

Note: Please don’t change the calibration options on the meter. If you have a concern, let me know.

Before Monitoring

Short questions:

Date:

Source name(s):

Site(s):

Name of contact:

Role of contact:

For how long has he or she had this role?

Telephone number for contact:

Gender of contact:

Will the site(s) be accessible for sampling even when the contact is not around?

Long-form answer questions:

What is the water from this supply used for?

What are the main challenges / problems for the water supply according to them?

Is the water good for drinking?

Do they have any concerns or areas of interest related to water quality?

Does the quality of the water change over time?

Is there any water treatment currently or has there ever been water treatment for this supply?

Has the water quality ever been tested? If yes, who did it? Were results reported? What were the result?

Have they been provided with information about water safety before?

DO NOT PROMPT, but please record if:

They asked you about payment for the water quality tests.

They asked you for something (advice, treatment help, more specific information).

After First Report

Short questions:

Date:

Site(s):

Did you take a photo of the results form?

Is anyone besides the primary contact listening or part of the conversation? (names and roles)

What language did you do the explanation and discussion in?

Were they attentive for the whole explanation and discussion or did they lose interest?

Are they happy for you to continue sampling?

Are they happy with the results to be reported over the phone in the future?

Long-form answer questions:

How did they react to the results (emotions, expression)?

What did they say about the results?

What did they say about the water treatment and safe storage information?

What did they said about the way the results have been reported?

Do they have any questions? Please record what they have asked you:

MONTHLY CHECK-INS

During the site visit:

Did you observe any changes since your last visit to the water supply?

Did any users tell you updates on problems or management activities for the water supply?

Did the primary contact tell you updates on problems or management activities for the water supply?

Did another committee or staff person tell you updates on problems or management activities for the water supply?

Did anybody ask you questions?

Please record any other comments from users or managers about the water safety.

During the result reporting:

Was reporting in-person or over the phone?

What was their emotional reaction to the result?

What did they say about the result?

(unprompted) Did they ask you any questions?

(unprompted) Did they express any intentions to act?

(unprompted) Did they give any explanations for not acting?

(follow-up) Have they implemented previously reported intentions?

(follow-up) Have they had challenges in attempting to implement previously reported intentions?

Did you discuss any water management topics other than water safety (e.g., maintenance issues, tariff issues, etc.)

END OF 2019

Short questions:

Date:

Site(s):

What was their level of satisfaction on repair and maintenance of the water supply?

- Extremely satisfied/excellent
- Very satisfied/very good
- Moderately satisfied/good
- Unhappy/poor
- Dissatisfied/very bad

Long-form answer questions:

What are the main challenges / problems for the water supply according to them?

What do they say about the goodness of the water for drinking?

Do they have any (other) concerns or areas of interest related to water quality?

(unprompted) In expressing this concern do they mention the monitoring and / or something else?

Were water treatment activities or measures to protect the water from contamination done in 2019? What was done? What were the reasons? Will this activity happen in 2020?

(unprompted) Have they given example(s) of a decision that was influenced by the monitoring results or water safety information?

(only for registered sites) Do they want the monitoring programme to continue?

(only for registered sites) Do they have suggestions for making the monitoring programme more useful? (Note: if they say they want increased support, please ask for details).

Do they have any questions? Please record what they have asked you:

MID 2020

Date:

Site(s):

How did they react to the water quality results?

Happy; Surprised; Sceptical (disbelieving); Worried; Distressed; Other [explain]

What have they said about the results?

Does the water taste fine for drinking? Does the water smell good for drinking? Is the colour of the water good for drinking? Are there any other reasons that people might not use this water for drinking?

Cost; Distance; Sick; Contaminated; Reliability; Other [explain]

Has there been any activity to protect the water from contamination or to clean it?

If yes, yow many different activities have been done? (loop)

If yes, is this a construction or installation activity? (such as building a fence or installing a filter for example)
If yes, what caused or motivated this activity to happen?
If yes, how was this funded?
If yes, was there any noticeable change about the water after doing the activity?
If yes, how often do they estimate this activity will continue to happen and why?

Are any (or any additional) treatment or protection activities being considered?
If no, what are the reasons?
If yes, what activities are being considered?
If yes, for how long have they thought of doing this?
If yes, what has discouraged them from doing the measure(s) already?
Cost; Sourcing materials; Know-how; Busy with other things; Other [explain]

What do they think about adding chlorine-based disinfectants to the water?
Good idea to make water safer; Worried about adding chemicals; Not interested; Other [explain]

Are they interested in hearing about options of disinfectant products that FundiFix may be able to sell to them?
If no, what are the reasons?
Unnecessary; Experience [explain]; Cost; Side effects; Unpleasant taste / smell; Other [explain]
If yes, do they think it would be useful if FundiFix sold any of the four options?
Yes; Unsure; No

If yes, which would they consider purchasing?
WaterGuard; Aquatabs; bulk chlorine; chlorine dispenser; none; unsure
If unsure, why?
Insufficient information [explain]; Time to think about it; Discuss with others first; Other [explain]

Do they have any other question or comments?

Supplementary File S4: LWM Interview Guide

Follow-up questions based on survey responses to integrate during the interview:

- …
- …

Stage	Question	Prompts
Overview	Can you tell me a bit about how [-] water supply is managed?	Who is involved?<br>What are the priority activities?
	What is your role?	Overview of responsibilities.<br>For how long have you been in this position?
	Have there been any break downs or big issues recently?	
	Can you tell me about the water quality monitoring programme?	
Water safety perceptions	Can you tell me about the quality of the water?	What do you personally think about it?<br>What do other managers think about it?<br>What do water users think about it?<br>How does it compare with other sources in the area?
	Can you tell me about your knowledge of water quality in general?	Where / from who have you learned about water quality?<br>Do they teach this topic in school?
	Have you received information from the water quality monitoring programme? Can you tell me about it?	What have they told you?<br>What else would you like to know?<br>Would you like to know test results from other sources? Which ones and why?
Generation of monitoring data	I understand you permit the monitoring programme to proceed. What are your thoughts about the way the work is done?	Your opinion? What do other managers think? Have users noticed the monitoring work? Do people talk about it? What do they say?
Engaging with monitoring data	Can you tell me what the results of the monitoring have been so far?	Check if they have kept the copy of the first report.
	How did you feel about these results?	If appropriate ask if / how feelings have changed over the last 6 months.
	Has receiving this information caused you to do anything differently?	Differentiate intentions and action.<br>Ask about rationale for acting or not.<br>Prompt discussion of source protection, treatment, and storage set-up and modification options as necessary.
Sharing monitoring data	Are the water quality results from your supply reported to anyone else by FundiFix?	Should they be?
	Have you shared the data with anyone? What were your reasons for sharing or not sharing?	Users? Other managers? Government? NGOs?
Responding to monitoring data	Who is responsible for responding to water quality problems?	What is your personal responsibility?<br>What are the roles of the users, the managers, FundiFix, NGOs, the government at 3 levels?
	Can you tell me about your interactions with the different groups that are involved in water supply?	Positive associations? Tensions?

Anything we haven't discussed that you think I should know / want to talk about?
Any questions for me?

First Reporting Form

This report is for:

Date:

**Site Name**	**December *E. coli* Result**

Water Quality Testing Information

There are three main types of water quality concerns:

1. Waterborne diseases from bacteria (e.g. cholera, typhoid), viruses, protozoa (e.g. amoeba) and worms;
2. Chemical components (e.g. high salinity);
3. Observable quality such as colour, smell, taste, turbidity.

For this report we are focussing on waterborne diseases. When water is contaminated with human and/or animal waste (faeces) it can contain types of bacteria, viruses, protozoa, and worms that may cause disease in humans. For example, diarrhoea and vomiting can be caused by drinking contaminated water. *E. coli* are bacteria that are very common in human and animal waste, so if *E. coli* is found in water it means that water might be contaminated and could cause waterborne diseases. The more *E. coli* in the water, the higher the chance that drinking the water could cause sickness.

The *E. coli* sampling results are reported as low, intermediate, high, or very high risk of waterborne disease:

- **Low risk** = no *E. coli*, low chance to cause waterborne disease.
- **Intermediate risk** = 1-10 *E. coli* in 100mL, may cause waterborne disease.
- **High risk** = 11-100 *E. coli* in 100mL, high chance to cause waterborne disease.
- **Very high risk** = more than 100 *E. coli* in 100mL, very high chance to cause waterborne disease.

The most common sources of faecal pollution are 1) unsafe management of wastewater and solid waste (e.g. open defecation (not using latrines), allowing livestock to defecate near water sources, building latrines too close to water sources, or not properly covering or fencing water sources to protect them from contact with faeces) and 2) swimming and/or bathing in water sources.

Water Treatment and Safe Storage Information

Bacteria (e.g. cholera, typhoid), viruses, and protozoa (e.g. amoeba) are not visible, so even if water looks clear it could still cause waterborne diseases. There are ways to protect, treat and store water to avoid waterborne diseases.

1. Source protection

Source protection means **keeping human and animal waste out of water**. This requires communities to work together. The first thing to do is identify the possible ways that water can be contaminated and then avoid it. For example:

- Keep the area around the water source clean so that no human or animal waste can be spread into the water;
- For rock catchments and earth dams, don't let humans or animals enter the water;
- Build latrines away from and downhill of water sources;
- Use latrines and avoid open defecation especially near water sources;
- Build fences to prevent animals from going near water sources;
- Build good drainage channels around taps and pumps so that water does not form surface ponds that can spread contamination down into the groundwater;
- Use concrete to cover the area around handpumps and wells so that contaminated surface water cannot flow directly into the groundwater; and
- Use clean containers to collect water.

2. Sedimentation

Even if you have done your best to protect the water, it may still contain contaminants that could cause waterborne diseases. **If the water is not clear, you can do sedimentation.** This is when you let water sit in a container and the particles that are suspended (floating) in the water slowly fall/settle out to the bottom. It is possible to add particular chemicals (e.g. alum or moringa) to the water to make the particles group together into clumps. The clumps are heavier, so they settle out faster. If you do not have these chemicals, you can still let sedimentation happen – it will just take longer. Put the water in a clean container, cover it so that no additional contaminants can enter, and wait. After some time, the water will become clearer. **Sedimentation is most important if you are doing filtration (so the filter doesn't block up) or doing disinfection with chlorine or UV (which work better if the water is clear).**

3. Filtration

After sedimentation, filtration can further increase the safety of water. Filtration involves passing water through a material that **catches bacteria, protozoa and worms and holds them back while clean water drains through.** There are many kinds of filters. Ceramic, for example, is often used as a filter material. Most filters need to be replaced or washed after some time so that they continue to work well.

4. Disinfection

Disinfection means **killing any bacteria, viruses, protozoa and worms that remain in the water**. There are different ways to disinfect. The most common are:

- Boiling – Boiling should be done for at least 1 to 3 minutes to be safe.
- Chlorine – Guidelines are available to know how much chlorine should be used for different volumes of water (e.g. WaterGuard and Aquatabs come with instructions for how much to use).
- Ultraviolet radiation exposure – Ultraviolet (UV) radiation comes from the sun. Water that is left in the direct sun for long enough can be disinfected by UV radiation. Some companies make special containers for this, but it is also possible to use clear plastic water bottles. It is important to do sedimentation and/or filtration first because pathogens can hide behind turbidity in the water to survive the UV exposure. It is also important to make sure the water is exposed to the sun for a long enough time. When the sun is strong, the water should be exposed for one day – including the hottest hours in the middle of the day. When the sun is not strong (e.g. there are clouds), the water should be exposed for two days. When it is raining, this method of disinfection should not be used because there is not enough UV radiation.

5. Safe storage

The final step is to store the water safely. Safe storage **makes sure that clean water stays clean and doesn't get contaminated while in storage.** Here are some other tips:

- Keep water in a clean and covered container;
- Remove water from the container using a tap or by pouring it through a narrow opening in a way that will not let anything get into the container and prevents hands from touching the water;
- Make sure the container is stable so that it does not tip over and if possible, keep it up off the ground;
- When the container is empty of water, clean it (with soap if possible) before refilling.

END OF YEAR REPORTING FORM

2019 Water Quality Monitoring Report (with example data)

Sampling Site	2018		2019										
	N	D	J	F	M	A	M	J	J	A	S	O	N
		H	L	L	L	L	L	L	L	L	L	L	L
		L	L		L	L	L		In	L	L		
		vH	H	H	H	H	vH		H	H			vH

For this report we are focusing on waterborne diseases.

When water is contaminated with human and/or animal waste (faeces), it can contain types of bacteria, viruses, protozoa, and worms that may cause disease in humans. For example, diarrhoea and vomiting can be caused by drinking contaminated water. *E. coli* are bacteria that are very common in human and animal waste, so if *E. coli* is found in water it means that water might be contaminated with faeces and could cause waterborne diseases. The more *E. coli* in the water, the higher the chance that drinking the water could cause sickness. The amount of *E. coli* in water can change because of rainfall, breakages, or other causes. That is why we took samples multiple times over the year.

The *E. coli* sampling results are reported as low, intermediate, high, or very high risk of waterborne disease:

Low risk	L	No *E. coli* detected, low chance to cause waterborne disease.
Intermediate risk	In	1 to 10 *E. coli* in a cup of water (100mL), may cause waterborne disease.
High risk	H	11 to 100 *E. coli* in a cup of water (100mL), high chance to cause waterborne disease.
Very high risk	vH	More than 100 *E. coli* in a cup of water (100mL), very high chance to cause waterborne disease.

The Kenyan Bureau of Standards and the World Health Organisation recommend that there should be no *E. coli* in drinking water. To avoid waterborne diseases, water that has *E. coli* in it should be disinfected by boiling or adding treatment such as WaterGuard or Aquatabs before it is consumed.
