# Supplementary material for: Fear, Efficacy, and Environmental Health Risk Reporting: Complex Responses to Water Quality Test Results in Low-Income Communities
Source: Int J Environ Res Public Health. 2022 Jan 5;19(1):597. doi: 10.3390/ijerph19010597 (PMC8744942; doi:10.3390/ijerph19010597)
Supplement: Supplementary file 1 [file ijerph-19-00597-s001.zip › Supplementary_Tables.pdf]

Supplementary Table S1: Integrated fear appeal framework concepts assessed through LWM surveys.

Survey	Description	Baseline Awareness	Perceived Threat	Perceived Efficacy	Affective State*	Intentions	Actions	Defensive Response
(1) before monitoring	Assessed perceptions of water safety prior to commencing monitoring, it included questions on water use, efforts to manage water cleanliness, and prior exposure to water quality testing or water safety information.	X	X	X				X
(2) after first report	Conducted immediately following the first sharing of *E. coli* results, it focused on capturing the LWMs' affective and cognitive responses to the information.		X	X	X	X		X
(3) monthly check-ins	Recorded observations and conversations from site visits and results-reporting with attention to affect display, stated intentions, questions, and actions of the LWMs in response to the monitoring results. Each sharing of results brings the LWM back to the message processing (undecided) stage and the short-term outcomes are a) stay undecided, b) no decision and move to uninvolved, c) intend to act (or continue acting), or d) intend no action.		X	X	X	X	X	X
(4) end of 2019	Conducted after the final reporting for 2019. LWMs were invited to reflect on the set of results, the utility of monitoring, and their experience with implementing water safety measures, where relevant.		X	X		X	X	X
(5) mid-2020	Supplies that were registered for maintenance services in 2020 continued to be monitored quarterly and a final survey of perceptions and intentions was conducted mid-year.		X	X	X	X	X	X

* measured by affect display

Supplementary Table S2: Percentages of LWMs reporting water safety intentions and actions at least once.

Approach	Activity	CBM		Facility		Private	
		Intended	Acted	Intended	Acted	Intended	Acted
Taking direct action	Treat water	25	11	87	60	50	17
	Clean tank(s)	39	18	53	40	0	0
	Install fencing	36	25	7	7	33	17
	Other source protection	18	14	27	20	50	33
	Implement source switching	0	0	40	33	0	0
Requesting support	Seek government support	18	11	7	7	0	0
	Seek NGO support	0	0	40	27	0	0
	Inform activism	4	0	0	0	0	0
Advising users to act	Instruct source protection	18	7	20	20	33	17
	Instruct HHWT	46	25	7	0	50	33
	Instruct source switching	0	0	7	7	0	0
	Instruct HHWT for other sources	18	11	40	20	17	17
Any	All	82	71	93	87	67	67

Supplementary Table S3: Defensive responses indicated at least once by LWMs following monitoring reporting.

**Defensive response**	**Description of expression.**	**No. of LWMs**
Downward comparison	Contextualising the reported monitoring results with reference to less protected drinking-water sources and other more severe threats.	21
Fatalism	Expressed by statements indicating that microbial contamination is natural or God's will, and is outside of the LWM's responsibility and ability to control.	11
Resignation	Indicated by statements of patience and waiting for efficacy to change through receipt of external support.	11
Reframing	Used for justification of inaction, for example by framing *E. coli* presence as a consequence of short-term, anomalous events like breakdown repairs.	10
Denial	Expressed by characterising test results as false alarms and presenting reasons why they may not be reliable, including no consequent cases of illness or no convincing pathway for *E. coli* ingress into the system.	8
Avoidance	One LWM withdrew permission for sampling to continue into 2020. Six other LWMs indicated avoidance by a combination of purposefully not sharing results with other committee members or administrators, low responsiveness to phone calls and meeting requests, and voicing that test results without solutions are unhelpful.	7
